# Dysregulation of microRNAs in breast cancer and their potential role as prognostic and predictive biomarkers in patient management

**DOI:** 10.1186/s13058-015-0526-y

**Published:** 2015-02-18

**Authors:** Eleni van Schooneveld, Hans Wildiers, Ignace Vergote, Peter B Vermeulen, Luc Y Dirix, Steven J Van Laere

**Affiliations:** 1 Department of Oncology, University Hospitals Leuven and Catholic University Leuven, Herestraat 49, Leuven, B-3000 Belgium; 2 CORE, Faculty of Medicine and Health Sciences, University of Antwerp, Universiteitsplein 1, Antwerp, B-2016 Belgium

## Abstract

MicroRNAs (miRNAs) are an emerging class of gene expression modulators with relevant roles in several biological processes, including cell differentiation, development, apoptosis, and regulation of the cell cycle. Deregulation of those tiny RNA molecules has been described frequently as a major determinant for the initiation and progression of diseases, including cancer. Not only miRNAs but also the enzymes responsible for miRNA processing could be deregulated in cancer. In this review, we address the role of miRNAs in the pathogenesis of breast cancer, since there are oncogenic, tumor-suppressive, and metastatic-influencing miRNAs. Additionally, the different detection platforms and normalization strategies for miRNAs will be discussed. The major part of this review, however, will focus on the capability of miRNAs to act as diagnostic, predictive, or prognostic biomarkers. We will give an overview of their potential to correlate with response to or benefit from a given treatment and we will consider their ability to give information on prognosis in breast cancer. We will focus on miRNAs validated by more than one study or verified in independent cohorts or where results rely on preclinical as well as clinical evidence. As such, we will discuss their potential use in the personalized management of breast cancer.

**Electronic supplementary material:**

The online version of this article (doi:10.1186/s13058-015-0526-y) contains supplementary material, which is available to authorized users.

## Introduction

### Breast cancer and breast cancer heterogeneity

Breast cancer is the most frequent carcinoma and second most common cause of cancer-related mortality in women [1]. Its heterogeneous character is reflected in the classification into four intrinsic subtypes (luminal A, luminal B, basal-like, and ErbB2^+^), a normal-like group, and a new subtype, referred to as claudin-low. Histologically, breast cancer can be divided into *in situ* and invasive carcinoma, both of which can be further subdivided into ductal and lobular. Stratification by integrative clustering is based on genomic and transcriptomic data [2]. Curtis and colleagues [2] revealed new biological subgroups as integrative clusters, characterized by well-defined copy number alterations, gene expression, and distinct clinical outcomes.

### MicroRNAs

MicroRNAs (miRNAs) are a group of small non-coding RNAs able to regulate gene expression at the post-transcriptional level by binding to the 3′ untranslated region (UTR) of target mRNAs. This leads to cleavage of the target mRNA by the Ago2 ribonuclease in the RNA-induced silencing complex (RISC) or inhibition of translation [3]. Alternatively, miRNAs can exert their effect by modulating the relationship between effector and target mRNAs rather than acting as regulators of particular mRNAs [4]. Deregulation of miRNA expression and potential altered gene expression may contribute to the development of cancerous phenotypes [5]. Several studies showed a differential miRNA expression profile in cancer as compared with normal, and a global miRNA downregulation was a common trait of human malignancies [6,7].

## Materials and methods

Relevant published articles were searched on PubMed databases from 2001 to present by using the following search criteria to retrieve articles and abstracts: (microRNA* or miR*) AND (breast cancer); depending on the chapter discussed in the review, we complemented this with AND (diagnostic OR prognostic OR predictive).

We focused on recent publications or findings validated by several independent studies. Studies were considered eligible on the basis of the following inclusion criteria: (i) miRNAs were evaluated in breast cancer cells or samples (blood or tissue) derived from patients with breast cancer, including publically available breast cancer cohorts; (ii) the relationship between the studied miRNAs and breast cancer biology was investigated, or (iii) the relationship between miRNAs and outcome or therapy response in breast cancer was examined. Articles were excluded if they met one of the following criteria: (i) published articles were retracted articles or comments; (ii) lack of key information on breast cancer biology, prognosis, or prediction of therapy response; and (iii) manuscripts reported on cancer types other than breast cancer.

## MicroRNAs and breast cancer

### The role of microRNAs in breast cancer biology and metastasis

In cancer, miRNAs play a role in oncogenesis, metastasis, and resistance to various therapies and can be classified as oncogenes (oncomirs) or tumor-suppressor genes [8-10]. Additionally, both pro-metastatic (‘metastamiRs’) and metastasis-suppressor miRNAs can be identified [11,12].

Many miRNA genes are located in genomic regions involved in chromosomal alterations. Chromosomal regions encompassing oncogenic miRNAs may be amplified, resulting in increased expression of the oncomir. Tumor-suppressive miRNAs could reside in fragile sites characterized by deletions or mutations, leading to reduced levels of these miRNAs [13,14]. An overview of the most prominent miRNAs involved in the pathogenesis of breast cancer is depicted in Tables 1 and 2.

**Table 1:** **List of major oncogenic microRNAs in breast cancer**

**miRNA**	**Target**	**Function**	**Number of samples** ^**a**^	**Reference**
**miR-10b**	HOXD10	Promotes cell migration, invasion and metastasis	n = 23	[8,16,17]
**miR-21**	PDCD4, HIF1A	Promotes invasion, metastasis, migration and EMT	Cell culture study	[18,19,22]
	TPM1, PTEN, PDCD4	Promotes invasion	n = 17	[20]
	TIMP3	Promotes invasion	n = 32	[21]
miR-155	SOCS1	Promotes cell growth and proliferation	n = 15	[23]
	TP53INP1	Promotes proliferation	Cell culture study	[24]
	FOXO3	Promotes proliferation and survival	n = 115	[25]
**miR-373**	CD44	Promotes cell migration and invasion	n = 11	[26]
		Promotes invasion and metastasis	Cell culture study	[27]
**miR-520c**	CD44	Promotes cell migration, invasion and metastasis	n = 11	[26]

MetastamiRs are indicated in bold. ^a^When applying more than one reference, we focused on the study with the most relevant number of investigated patient samples. The other studies serve as validation and confirm the results. EMT, epithelial-to-mesenchymal transition; FOXO3, forkhead box protein O3; HIF1A, hypoxia-inducible factor-1α; HOXD10, homeobox D10; miRNA, microRNA; PDCD4, programmed cell death protein 4; PTEN, phosphatase and tensin homolog; SOCS1, suppressor of cytokine signaling 1; TIMP3, metalloproteinase inhibitor 3; TM1, tropomyosine 1; TP53INP, tumor protein p53 inducible nuclear protein.

**Table 2:** **List of major tumor suppressive microRNAs in breast cancer**

**miRNA**	**Target**	**Function**	**Number of samples** ^**a**^	**Reference**
**miR-125b**	EPO, EPOR	Inhibits cell proliferation and differentiation	n = 42	[29]
	ENPEP, CK2-α,CCNJ, MEGF9	Inhibits cell proliferation	n = 25	[30]
	ERBB2	Inhibits migration and invasion	Cell line study	[31]
miR-205	HMGB3	Suppresses proliferation and invasion	n = 20	[32,33]
**miR-17-92**	Mekk2	Promotes NK cell antitumoral activity and reduces metastasis	n = 20	[34,35]
**miR-206**	Cyclin D2, Cx43	Reduces migration, invasion and metastasis	n = 128	[36,37]
**miR-200**	ZEB1/2, SNAI1/2	Reduces tumor growth, metastasis and EMT	n = 70	[38-40]
**miR-146b**	NFkB, STAT3	Reduces survival and metastasis	n = 91	[41,42]
**miR-126**	IGFBP2, MERTK,PITPNC1	Reduces metastasis and angiogenesis	n = 117, n = 295	[43]
	No specific targets listed	Reduces tumorigenesis and metastasis	Cell line study	[44]
**miR-335**	SOX4, TNC	Suppresses metastasis and migration	n = 20	[44,45]
**miR-31**	RhoA	Inhibits several steps of the invasion-metastasis cascade in breast cancer	n = 54	[46]
	WAVE3, RhoA	Reduces cancer progression and metastasis	Cell line study	[47]
	WAVE3	Reduces cancer progression and metastasis	n = 19	[48]

Metastasis-suppressive miRs are indicated in bold. ^a^When applying more than one reference, we focused on the study with the most relevant number of investigated patient samples. The other studies serve as validation and confirm the results. CCNJ, cyclin J; CK2-α, casein kinase 2-alpha; Cx43, connexin 43; ENPEP, glutamylaminopeptidase or aminopeptidase A; EPO, erythropoietin; EPOR, erythropoietin receptor; ERBB2, Receptor tyrosine-protein kinase erbB-2 (human epidermal growth factor receptor 2); HMGB3, high-mobility group box 3 gene; IGFBP2, insulin-like growth factor-binding protein 2; MEGF9, multiple EGF-like domains 9; Mekk2, mitogen-activated protein kinase kinase kinase 2; MERTK, c-Mer tyrosine kinase; miRNA, microRNA; NFkB, nuclear factor kappa B; NK, natural killer; PITPNC1, phosphatidylinositol transfer protein, cytoplasmic 1; RhoA, Ras homolog gene family; SNAI1/2, snail family zinc finger 1/2; SOX4, SRY-related HMG-box 4; STAT3, signal transducer and activator of transcription 3; TNC, tenascin C; WAVE3, WAS protein family, member 3; ZEB1/2, zinc finger E-box binding homeobox 1/2.

#### Oncogenic microRNAs and ‘metastamiRs’

Examples of breast oncomirs are miR-10b, miR-21, miR-155, miR-373, and miR-520c. (See Table 1 and list of abbreviations for miRNA targets.) OncomiRs exert their oncogenic activity by targeting tumor-suppressor genes and activating oncogenic transcription factors [8,15].

MiR-10b targets HOXD10, thereby promoting cell migration and invasion [8,16,17]. miR-21 has been reported to be associated with invasive and metastatic breast cancer [18] and regulates epithelial-to-mesenchymal transition (EMT) and HIF1A in breast cancer stem cell-like cells [19]. It also inhibits various tumor-suppressor proteins [20-22]. MiR-155 suppresses SOCS1 both *in vivo* and *in vitro* [23], and upregulation results in proliferation of MCF7 cells [24]. FOXO3 was identified as another miR-155 target, whose inhibition leads to enhanced cell survival and tumor growth [25]. The expression of miR-373 and -520c, which target CD44, is associated with metastasis, invasion, and migration [26,27].

#### Tumor-suppressive and metastasis-suppressive microRNAs

Tumor-suppressor miRNAs exhibit a lower expression in cancer cells and suppress oncogene expression, thereby controlling cellular differentiation [28]. In regard to the global downregulation of miRNAs in cancer, it is worth mentioning that the majority of miRNAs have a tumor-suppressive function, some of which exhibit anti-metastatic properties as well. (See Table 2 and list of abbreviations for miRNA targets).

MiR-125b, which targets erythropoietin (EPO) and its receptor (EPOR) as well as ERBB2, is found to be one of the most downregulated miRNAs in breast cancer [29-31]. MiR-205 regulates HMGB3 and its ectopic expression significantly inhibits cell proliferation and promotes apoptosis in breast cancer [32,33]. Jiang and colleagues [34] revealed how expression of the miR-17-92 cluster in triple-negative breast cancer (TNBC) was associated with significantly reduced metastasis [35]. miR-206 inhibits cell proliferation, migration, and invasion by targeting cyclin D2 and Cx43 [36,37].

Certain tumor-suppressive miRNAs have an inhibitory role in the metastasis cascade. For example, the miR-200 family regulates EMT via E-cadherin expression by targeting several EMT inducers [38-40]. MiR-146b inhibits nuclear factor kappa B (NF-κB)-dependent interleukin (IL)-6 expression, which is associated with impaired survival and metastasis of cancer cells [41,42]. Decreased expression of miR-126 was seen in a variety of human cancers, and restoration of expression reduced metastatic endothelial recruitment and angiogenesis [43,44]. MiR-335 is known to target several metastasis-associated genes (for example, *SOX4* and *TNC*) [44,45]. Finally, independent studies have reported the pleiotropic actions of miR-31 on breast cancer metastasis and its ability to suppress multiple steps of the invasion-metastasis cascade, by targeting RhoA and WAVE3 [46-48].

#### Context-dependent microRNAs

The action of certain miRNAs is dependent upon cellular or environmental context and results in both tumor-suppressive and -promoting roles [17,49]. This could in part explain possible inconsistent outcomes from distinct miRNA-examining studies.

As described above, miR-146 exerts its tumor-suppressive action through negative regulation of NF-κB signaling [41]. However, an additional target is the pro-apoptotic DNA repair enzyme BRCA1 [50]. MiR-29 stimulates metastasis and EMT by suppressing the cell-adhesion molecule peroxidasin homologue but is also capable of restraining cancer progression by targeting proliferation-inducing oncogenes, suppressing DNA methylation of tumor-suppressor genes, and increasing chemosensitivity [51]. Although most data support an oncogenic function for miR-373 and -520c [26,27], a recent study reported this miR-family as a tumor suppressor in estrogen receptor (ER)-negative breast cancer [52].

## MicroRNA detection and normalization

### MicroRNA detection and genome-wide approaches

Many hybridization-based miRNA detection platforms are being described. Though low-throughput, the most standardized method for miRNA analysis is Northern blotting [53]. High-throughput methods for miRNA detection are the oligonucleotide miRNA microarray and real-time reverse transcription-polymerase chain reaction (RT-PCR). MiRNA microarrays are capable of analyzing hundreds of miRNAs (genome wide) in a large number of samples. Integration of the obtained miRNA data with mRNA expression data is useful to discover new miRNA-gene interactions [54]. RT-PCR is another sensitive technique used for assessment of miRNA expression or validation of data obtained from other detection platforms [55]. Other detection techniques include bead-based flow cytometry [6] and *in situ* hybridization [56]. The limitation of the above-mentioned platforms is their restriction to known miRNA structures. Next-generation sequencing technologies provide new approaches for discovery of new and confirmation of known miRNAs [57,58]. An overview of the most prominent miRNA detection methods is shown in Table 3.

**Table 3:** **The most prominent microRNA detection methods**

**Detection method**	**Throughput**	**Sensitivity**	**Specificity**	**Reference**
Northern blotting	Low	Low	High	[53]
Microarray	High	Low	Low	[54]
Bead-based flow cytometry	High	Medium	High	[6]
qRT-PCR	High	High	High	[55]
*In situ* hybridization	Low	Low	Low	[56]
Next-generation sequencing	High	High	High	[57,58]

qRT-PCR, quantitative real-time polymerase chain reaction.

### MicroRNA normalization

Good normalization procedures for miRNA expression are still lacking. A normalization strategy for miRNA quantitative RT-PCR (qRT-PCR) is the use of endogenous controls, like reference miRNAs (for example, miR-16) or other small non-coding RNAs (for example, small nuclear and nucleolar RNA). However, it is recommended to use reference molecules belonging to the same RNA class since they possess the same physicochemical properties [59]. This is why miR-16 would be preferable over the use of small nucleolar and nuclear RNAs and was used in our study to normalize quantitative PCR data obtained from miRNA expression analysis in blood [60]. Also, the quantification accuracy increases by use of more than one reference gene [59]. Another example of a potential reliable reference gene is miR-484, whose reference-gene utility may be enhanced by averaging with other potential breast cancer housekeeping miRNAs (for example, miR-330-3p, -4327, and -1271) [4]. Other normalization strategies for miRNA expression include the mean expression value strategy, as proposed by Mestdagh and colleagues [61].

## MicroRNAs as diagnostic, predictive, and prognostic biomarkers

**Figure 1:**
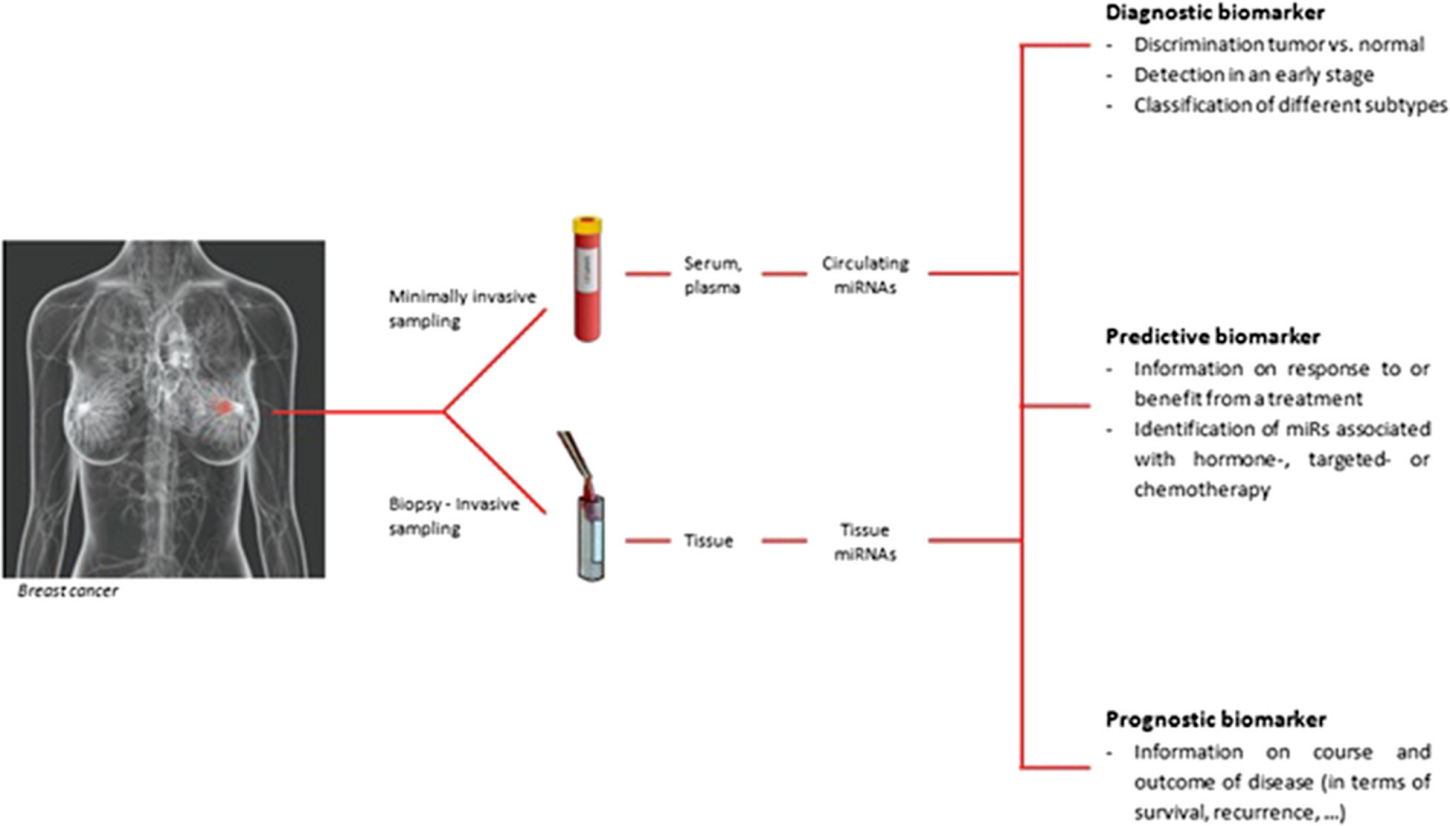
**Illustration of the application of microRNAs (miRNAs) as novel diagnostic, predictive, and prognostic biomarkers in breast cancer management.**

The ideal biomarker should be easily accessible, sensitive enough to detect all tumors, and specific and therefore not detectable in healthy individuals. Because of their high tissue-specificity, great stability, and aberrant expression in different tumor types, miRNAs are thought of as specific biomarkers with diagnostic, predictive, and prognostic potential (Figure 1).

### MicroRNAs as diagnostic biomarkers for breast cancer heterogeneity

#### Early diagnosis of breast cancer

Early diagnosis is of utmost importance to reduce the mortality rate in cancer. Therefore, the search for new diagnostic biomarkers remains a persisting quest. The main examined diagnostic miRNA signatures will be discussed, with a focus on those miRNAs validated by more than one study or verified in different cohorts or both. (For a detailed overview of the individual miRNAs, see Table 4).

**Table 4:** **List of major diagnostic microRNA signatures for the early diagnosis of breast cancer**

**miR signature**	**Expression (BC vs normal)**	**Sample type**	**Number of samples: BC (normal)**	**Validation**	**Reference**
miR-15a	Upregulated	Serum	n = 48 (24)	n = 60 (51)	[62]
**miR-18a**	Upregulated	Serum	n = 48 (24)	n = 60 (51)	[62]
miR-107	Upregulated	Serum	n = 48 (24)	n = 60 (51)	[62]
miR-425	Upregulated	Serum	n = 48 (24)	n = 60 (51)	[62]
**miR-133a**	Downregulated	Serum	n = 48 (24)	n = 60 (51)	[62]
miR-139-5p	Downregulated	Serum	n = 48 (24)	n = 60 (51)	[62]
miR-143	Downregulated	Serum	n = 48 (24)	n = 60 (51)	[62]
**miR-145**	Downregulated	Serum	n = 48 (24)	n = 60 (51)	[62]
miR-365	Downregulated	Serum	n = 48 (24)	n = 60 (51)	[62]
miR-155	Upregulated	Serum	n = 184 (75)	Meta-analysis (inclusion of 3 studies*)*	[63]
miR-1	Upregulated	Serum	n = 32 (22)	n = 132 (101)	[64]
**miR-133a**	Upregulated	Serum	n = 32 (22)	n = 132 (101)	[64]
miR-133b	Upregulated	Serum	n = 32 (22)	n = 132 (101)	[64]
miR-92a	Upregulated	Serum	n = 32 (22)	n = 132 (101)	[64]
miR-148b	Upregulated	Plasma	n = 127 (80)	n = 207 (80)	[65]
miR-376c	Upregulated	Plasma	n = 127 (80)	n = 207 (80)	[65]
miR-409-3p	Upregulated	Plasma	n = 127 (80)	n = 207 (80)	[65]
miR-801	Upregulated	Plasma	n = 127 (80)	n = 207 (80)	[65]
miR-16	Upregulated	Plasma & tissue	n = 15 (15)	n = 170 (100)^a^	[66]
miR-21	Upregulated	Plasma & tissue	n = 15 (15)	n = 170 (100)^a^	[66]
miR-451	Upregulated	Plasma & tissue	n = 15 (15)	n = 170 (100)^a^	[66]
**miR-145**	Downregulated	Plasma & tissue	n = 15 (15)	n = 170 (100)^a^	[66]
**miR-18a**	Overexpressed in cases compared to controls^b^	Serum	n = 205 (205)	n = 5 (5)	[67]
miR-181a	Overexpressed in cases compared to controls^b^	Serum	n = 205 (205)	n = 5 (5)	[67]
miR-222	Overexpressed in cases compared to controls^b^	Serum	n = 205 (205)	n = 5 (5)	[67]

Common microRNAs (miRNAs) are indicated in bold. ^a^miRs only detected in plasma, not in tissue. ^b^Cases, women who developed breast cancer (BC); controls, women who stayed BC-free.

In a recent study, a signature of nine circulating miRNAs was capable of discriminating between early-stage breast cancer and healthy controls [62]. A meta-analysis concerning miR-155 showed highly sensitive and specific diagnostic accuracy [63]. By comparing miRNA profiles between serum samples from breast cancer patients and healthy volunteers, Chan and colleagues [64] identified four miRNAs as significant diagnostic markers. Cuk and colleagues [65] found another four upregulated miRNAs in plasma of patients with breast cancer, capable of detecting stage I or II breast cancer, making them attractive candidates for early breast cancer detection. Another study reported three upregulated miRNAs and one downregulated miRNA in patients with breast cancer compared with normal controls [66]. A prospective study identified three significantly overexpressed serum miRNAs in women who eventually developed breast cancer (cases) compared with breast cancer-free controls [67]. This study introduces a new perspective on the miRNA function by describing their potential to predict increased risk of developing breast cancer.

#### Breast cancer molecular subtypes and microRNAs

We have shown that microRNA expression profiles are able to recapitulate the molecular breast cancer subtypes by using mRNA profiling [60,68]. Molecular miRNA signatures which distinguish between different breast cancer subtypes were described for the first time (to the best of our knowledge) by Blenkiron and colleagues [69] and reviewed by Serpico and colleagues [70]. The commonly found molecular subtype-related miRNAs are presented in Table 5.

**Table 5:** **Common subtype-specific microRNAs found by meta-analysis of three independent studies**

**Luminal A**	**Basal**	**HER2**	**Normal-like**
let-7c	miR-18a	miR-142-3p	miR-145
miR-10a	miR-135b	miR-150	miR-99a
let-7f	miR-93		miR-100
	miR-155		miR-130a

Common subtype-specific microRNAs (miRNAs)^a^ derived from analysis by Dvinge *et al*. [4] (2013), Blenkiron *et al.* [69] (2007), and de Rinaldis *et al*. [71] (2013). ^a^No common miRNAs for the luminal B subtype could be found.

Blenkiron and colleagues [69] profiled 309 miRNAs in 93 breast tumors with different molecular subtypes. The differential miRNA expression resulted in a correct classification of basal versus luminal subtypes. An identified 31-miRNA signature was able to distinguish the different breast cancer subtypes. de Rinaldis and colleagues [71] found consistent results by revealing a 46-miRNA signature able to differentiate between breast cancer subtypes (n = 173). A substantial degree of consistency was also observed by Dvigne and colleagues [4], and the common miRNA signatures for molecular breast cancer subtypes are listed in Table 5.

In another study, 453 miRNAs in 29 early-stage breast cancer tumors were profiled, identifying signatures that accurately predict ER, progesterone receptor (PR), and HER2 status (Table 6) [72]. miR-342 showed the highest expression in ER-positive and HER2/neu-positive luminal B tumors, which was verified in recent studies [73,74], and showed a decreased expression in TNBC. MiR-520 g was downregulated in ER- and PR-positive tumors.

**Table 6:** **MicroRNA signatures for estrogen receptor, progesterone receptor, and HER2/neu receptor status in breast cancer**

**miRNA**	**Median accuracy, percentage**	**Increased miRNA expression gives higher probability of:**
ER status		
miR-342	83.3	ER (+) status
miR-299-3p	100	ER (−) status
miR-217	100	ER (+) status
miR-190	100	ER (−) status
miR-135b	100	ER (-) status
miR-218	100	ER (+) status
PR status		
miR-520 g	83.3	PR (−) status
miR-377	83.3	PR (+) status
miR-527-518a	100	PR (−) status
miR-520f-520c	100	PR (+) status
HER2/neu status		
miR-520d	100	HER2/neu (+) status
miR-181c	100	HER2/neu (−) status
miR-302c	100	Weak response
miR-376b	100	HER2/neu (+) status
miR-30e-3p	100	Weak response

Lowery *et al*. [72] (2009). ER, estrogen receptor; miRNA, microRNA; PR, progesterone receptor.

#### Breast cancer histological subtypes and microRNAs

Little is known about the correlation of histological subtypes and specific miRNA expression patterns and it is definitely worthwhile to further investigate this relation in the future.

Volinia and colleagues [75] found a nine-miR invasiveness signature by profiling miRNAs in ductal carcinoma *in situ* and invasive ductal carcinoma (IDC). Giricz and colleagues [76] revealed six differentially expressed miRNAs during progression from lobular carcinoma *in situ* to invasive lobular carcinoma. Van der Auwera and colleagues [77] showed a differential expression of 13 miRNAs in inflammatory breast cancer (IBC) versus non-IBC. Another study examined the miRNA expression profile in IBC and found five miRNAs able to accurately classify between IBC and non-IBC [78].

### Predictive microRNAs

Predictors help to individualize therapy and diagnosis of breast cancer, correlate with response to a given treatment, and determine the treatment benefit. Recently, several miRNAs have been described to serve as putative therapeutic targets. (See Table 7 for described miRNAs and Additional file 1: Table SI 1 for a more extensive overview of predictive miRNAs).

**Table 7:** **Predictive microRNAs - microRNAs involved in response (sensitivity/resistance) to conventional breast cancer therapeutic strategies**

**Therapy**	**Generic name**	**miRNA**	**Role in response** ^**a**^	**Evidence**	**Number of patients or type of cells**	**Reference**
Hormone therapy						
SERM	Tamoxifen	miR-375	Sensitivity	Preclinical/clinical	2 BC datasets	[80]
		miR-342	Sensitivity	Preclinical	MCF-7	[74]
			Sensitivity	Clinical	n = 791	[81]
		miR-221/222	Resistance	Preclinical	MCF-7, T47D, MM-468	[82-84]
SERD	Fulvestrant	miR-221/222	Resistance	Preclinical	MCF-7	[85]
AI	Letrozole	let-7f	Sensitivity	Preclinical/clinical	n = 23	[86]
Targeted therapy						
Monoclonal AB	Trastuzumab	miR-210	Resistance	Preclinical/clinical	n = 43	[87]
Chemotherapy						
	FEC	miR-125b	Resistance	Preclinical/clinical	n = 56	[89]
			Resistance	Preclinical	MM-435, SKBR3	[88]
			Resistance	Clinical	n = 185	[90]
	Taxol/doxo	miR-30c	Sensitivity	Preclinical	T47D, MCF-7, MM-231	[91,92]
	Taxol	miR-21	Resistance	Preclinical	MM-468	[9,93]
Radiotherapy						
	Radiotherapy	miR-34a	Sensitivity	Preclinical	T47D, MCF-7, MM-231	[95,96]

^a^MicroRNA (miRNA) overexpression leads to an increase in resistance or sensitivity to the mentioned therapy (referred to as ‘resistance’ or ‘sensitivity’, respectively). AB, antibody; AI, aromatase inhibitor; BC, breast cancer; Doxo, doxorubicin; FEC, fluorouracil, epirubucin and cyclophosphamide; SERD, selective estrogen receptor downregulator; SERM, selective estrogen receptor modulator; Taxol, paclitaxel.

#### MicroRNAs associated with hormone therapies

MiRNAs appear to be involved in the process of endocrine resistance [79], and research has been conducted to identify miRNAs associated with clinical benefit of hormone therapies.

**miR-375** miR-375 demonstrated its sensitizing effect on tamoxifen response via direct targeting of metadherin (MTDH). Loss of MTDH restored sensitivity to tamoxifen and correlated with disease-free survival (DFS) in tamoxifen-treated patients [80].

**miR-342** He and colleagues [74] found that miR-342 expression positively correlates with ERα expression and that introducing miR-342 into estrogen-dependent breast cancer cell lines enhanced sensitivity to tamoxifen-induced apoptosis. Cittelly and colleagues [81] verified these findings and reported miR-342 downregulation to be associated with tamoxifen resistance.

**miR-221/222** The miR-221/222 cluster is an inhibitor of ERα and is being associated with tamoxifen resistance in breast cancer cells [82-84]. This miRNA cluster has also been involved in resistance to fulvestrant, a selective ER downregulator [85].

**Let-7f** Aromatase inhibitors are used in endocrine therapy since they decrease estrogen production by blocking the aromatase gene *CYP19A1*, a direct target of let-7 f. MiRNA expression profiling before and after treatment with letrozole showed an increase in let-7f in preclinical as well as clinical settings [86].

#### MicroRNAs associated with targeted therapies

**miR-210** Jung and colleagues [87] examined the plasma miR-210 expression level in patients with HER2^+^ breast cancer before (that is, baseline expression) and after neoadjuvant chemotherapy (NCT) that included trastuzumab. MiR-210 was the only miRNA with a significant upregulated baseline expression in the residual disease group before treatment. Therefore, high miR-210 baseline expression was associated with resistance to trastuzumab-included chemotherapy. Results were validated by comparing miR-210 expression in trastuzumab-sensitive and -resistant breast cancer cells and in an independent set of pre- and post-operative plasma samples. A significantly higher miR-210 level was found in the trastuzumab-resistant cells and in the independent patient cohort before surgery.

#### MicroRNAs associated with response to chemotherapeutic agents

Drug sensitivity varies with each patient, making predictors of benefit or resistance toward proposed chemotherapeutic agents essential. In this way, the proportion of patients with a beneficial treatment would increase and toxicity of ineffective treatments would be avoided.

**miR-125b** Zhou and colleagues [88] reported resistance to various chemotherapies induced by miR-125b targeted repression of Bak1 (BCL2 antagonist killer 1), which was verified by the clinical findings of Wang and colleagues [89], who reported higher miR-125b expression in non-responsive patients after admission of 5-fluorouracil. Climent and colleagues [90] reported miR-125b deletion on chromosome 11q to be correlated with benefit of anthracycline-based chemotherapy and low recurrence rate in patients with lymph node-negative breast cancer.

**miR-30c** Bockhorn and colleagues [91] described how increased levels of miR-30c sensitized the drug response of breast cancer cell lines to paclitaxel and doxorubicin, and this was also seen in the preclinical model by Fang and colleagues [92].

**miR-21** Mei and colleagues [93] reported miR-21 upregulation to be associated with taxol resistance in breast cancer cells. These results were validated in a recent study, in which miR-21 upregulation resulted in an increase of the anti-apoptosis protein BCL-2 and chemoresistance in breast cancer cells [9]. The increase of BCL-2 expression was induced by direct targeting by miR-21, which was able to unconventionally upregulate the expression of its direct target by binding to its 3′ UTR [94].

#### MicroRNAs associated with radiotherapy

**miR-34a** Low levels of miR-34a rendered breast cancer cells more resistant to radiotherapy [95]. These findings were verified by Stankevicins and colleagues [96], who postulated that miR-34a was involved in breast cell responses to low-dose X radiation. Moreover, miR-34a was found to be upregulated by p53 in response to radiation in normal and breast cancer cell lines [96].

### Prognostic microRNAs

Since patients at higher risk might acquire differential therapeutic interventions, the search for prognostic biomarkers remains a continuous work in progress. Several gene-expression studies have identified new or improved miRNA prognostic markers, giving information on the course and outcome of disease in different subgroups of patients. To narrow this extensive area, we will emphasize those prognostic miRNAs that are being validated (that is, examined in more than one study or tested in the same study) but verified in more than one cohort or dataset. An overview of the miRNAs described in this review can be found in Table 8 and Table 9; for a more extensive list, see Additional file 1: Tables S2A and S2B.

**Table 8:** **List of positive prognostic microRNA signatures in breast cancer**

**miRNAs associated with positive outcome**						
**miRNA**	**Case cohort**			**Validation cohort**		**Reference**
	**Detection**	**Cells of origin**	**Number of samples**	**Technique**	**Number of samples**	
let-7b	LNA-ISH	Early invasive BC	1,432	qRT-PCR	40	[97]
	LNA-ISH	Heterogeneous BC	80	NR	NR	[101]
miR-205	LNA-ISH	Ductal BC	1,475	qRT-PCR	40	[97]
	qRT-PCR	Heterogeneous BC	84	NR	NR	[102]
miR-375	Solexa deep sequencing	Stage II-III BC	42	qRT-PCR	26	[57]
miR-30a	miRNA microarray	IDC	221	NR	NR	[104]
	qRT-PCR	Heterogeneous BC	96	NR	NR	[108]
miR-342-5p	miRNA microarray	Heterogeneous BC	101	miRNA microarray	1,302	[109]
miR-497	qRT-PCR	Heterogeneous BC	128	NR	NR	[111]
	qRT-PCR	IDC	48	NR	NR	[110]

BC, breast cancer; IDC, invasive ductal carcinoma; LNA-ISH, Locked Nucleic Acid-*in situ* hybridization; miRNA, microRNA; NR, not reported; qRT-PCR, quantitative real-time polymerase chain reaction.

**Table 9:** **List of negative prognostic microRNA signatures in breast cancer**

**miRNAs associated with negative outcome**						
**miRNA**	**Case cohort**			**Validation cohort**		**Reference**
	**Detection**	**Cells of origin**	**Number of samples**	**Technique**	**Number of samples**	
miR-122	Deep sequencing	Heterogeneous BC	42	qRT-PCR	26	[57]
miR-27b-3p	qRT-PCR	TNBC	58	qRT-PCR	41	[113]
miR-21	qRT-PCR	IDC	109	NR	NR	[115]
	qRT-PCR	Heterogeneous BC	84	NR	NR	[102]
miR-210	Deep sequencing	IDC	118	NR	NR	[75]
	Meta-analysis	NR	699	Meta-analysis	Meta-analysis	[116]
	Meta-analysis	NR	1,809	Meta-analysis	Meta-analysis	[117]
miR-9	miRNA microarray	ER^+^ BC	16	qRT-PCR	52	[120]
miR-187	LNA miRCURY	LN^+^ BC	117	LNA miR probe	470	[121]
miR-155	qRT-PCR	Heterogeneous BC	88	NR	NR	[122]
	qRT-PCR	Heterogeneous BC	231	NR	NR	[123]

BC, breast cancer; ER, estrogen receptor; IDC, invasive ductal carcinoma; LN, lymph node; LNA, Locked Nucleic Acid; miRNA, microRNA; NR, not reported; qRT-PCR, quantitative real-time polymerase chain reaction; TNBC, triple-negative breast cancer.

#### MicroRNAs associated with positive prognosis

**Let-7b and miR-205** Quesne and colleagues [97] revealed association of let-7b and miR-205 with prognosis in breast cancer. They applied *in situ* hybridization to study miRNA expression in a population-based breast tumor cohort and validated their findings by use of qRT-PCR. Deregulation of let-7b, which targets the oncogenes *H-RAS* and *HMGA2*, often occurs early in breast cancer progression and its expression is known to be downregulated during EMT and associated with less aggressive tumors [69,98-100]. Within luminal breast cancer, increased let-7b expression was positively associated with survival.

Additionally, the authors reported miR-205 as another miRNA with positive prognostic value [97]. MiR-205 regulates EMT by inhibiting E-cadherin suppression. MiR-205 expression is associated with tumors of ductal morphology and independently predicts survival within this tumor subtype.

Two independent studies verified these results. Ma and colleagues [101] showed how low let-7b expression levels in a heterogeneous breast cancer cohort associated with poor prognosis reflected in lower overall survival (OS) and relapse-free survival (RFS) times. Markou and colleagues [102] detected miRNA-205 levels in a heterogeneous breast cancer cohort and demonstrated how downregulation was associated with longer DFS.

**miR-375** Wu and colleagues [57] applied deep sequencing methods on blood of patients with primary stage II or III breast cancer to indicate the miR-375 prognostic value and validated these findings in an independent cohort by using qRT-PCR. miR-375 is known to inhibit migration and invasion which is partially carried out by targeting JAK2 [103]. A differential expression exists between patients with and without metastatic relapse. Higher circulating levels reflect more favorable clinical outcome in terms of pathologic complete response to NCT and absence of relapse [57].

**miR-30a** The prognostic features of miR-30a were investigated in 221 patients with IDC [104], showing that miR-30a, which has been implicated in regulation of several biological processes [105-107], negatively regulates vimentin expression and overexpression suppresses migration and invasiveness [104]. Reduced expression is associated with unfavorable outcome (decreased RFS and DFS). The prognostic value of miR-30a was also examined by Zhang and colleagues [108] on a heterogeneous set of 96 patients with breast cancer. The authors attributed the tumor-suppressive nature of this miRNA to its ability to target MTDH, thereby suppressing breast tumor growth and metastasis. Decreased expression of miR-30a was associated with an unfavorable outcome in terms of metastasis development [108].

**miR-342-5p** miR-342-5p has a role in cell cycle progression and cell growth regulation. The latest research by Leivonen and colleagues [109] demonstrated how higher expression of miR-342-5p, which is an efficient negative regulator of the HER2 pathway, was significantly associated with better survival in two heterogeneous breast cancer cohorts.

**miR-497** miR-497 is a tumor-suppressive miRNA and its biological role is found in the regulation of the nervous system. Several studies examined miR-497 and found its expression to be significantly decreased in breast cancer compared with normal breast and negatively correlated with adverse clinicopathological characteristics. Shen and colleagues [110] reported how elevated miR-497 expression rendered IDC patients with better prognosis. An independent study verified its prognostic role in a population-based breast cancer cohort, in which higher expression was correlated with a better 5-year DFS and OS [111].

#### MicroRNAs associated with negative prognosis

**miR-122** Wu and colleagues [57] identified circulating miR-122 in a heterogeneous breast cancer exploration and validation cohort, by sequencing and RT-PCR, respectively. They found miR-122 to be associated with outcome in terms of relapse and identified a distinct expression in patients undergoing metastatic relapse and those who remained relapse-free.

**miR-27b-3p** The miR-27 family is known to regulate cell cycle progression and survival by targeting the tumor-suppressive FOXO1 gene and is highly expressed in breast cancer [112]. In a recent study, Shen and colleagues [113] aimed to identify and validate a prognostic signature for patients with TNBC (n = 58) and found that lymph node status and miR-27b-3p were independent predictors of poor prognosis in terms of distant metastasis-free survival. This result was validated in a TNBC cohort (n = 41) [113].

**miR-21** miR-21, which has its biological role in development, morphogenesis, and differentiation, is known to be overexpressed in breast cancer [114]. The significance of this miRNA as a prognostic factor was examined in two independent studies. Lee and colleagues [115] found significantly higher expressions in IDC compared with normal breast tissue, which positively associated with tumor size, stage, grade, and Ki-67 expression. A higher miR-21 expression also correlated with ER negativity and HER2 positivity. A lower OS could be noticed in patients with higher miR-21 expression levels [115]. In a more recent study, miR-21 was found to inversely correlate with DFS [102].

**miR-210** Volinia and colleagues [75] used deep sequencing to determine miR-210 in patients with IDC. miR-210 showed association with time to metastasis and OS by uni- and multivariate analyses. Various breast cancer genes—such as BRCA1, E-cadherin, PARP1, and RB1—with an antagonistic behavior to miR-210 were identified. More recently, systematic reviews and meta-analyses of previous clinical research were performed to recapitulate the significance of increased miR-210 in the prognosis of cancer. Summarizing nine published studies, the authors found that miR-210 could predict outcome, especially in patients with breast cancer, and that miR-210 overexpression predicts DFS and RFS [116,117].

**miR-9** The functional significance of miR-9 is evidenced by its regulative role in neurogenesis, development, and apoptosis. Dysregulation of miR-9 influences proliferation or metastasis formation and has been reported in cancer [118]. In breast cancer, upregulation of miR-9 suppresses E-cadherin, leading to increased cell motility and invasiveness [119]. Zhou and colleagues [120] found miR-9 to discriminate between cases with and without local recurrence (LR), in which the latter group showed a significantly lower expression. In ER^+^ cases, a lower 10-year LR-free survival was seen in patients with high miR-9 expression by using miRNA arrays [120].

**miR-187** Using an *in silico* method in two independent breast cancer cohorts, Mulrane and colleagues [121] identified and validated miRNA-187 as being involved in breast cancer progression. miR-187 is independently associated with poor outcome in breast cancer (particularly, lymph node-positive samples) in terms of reduced breast cancer-specific survival.

**miR-155** Several studies demonstrated the involvement of miR-155 in biological processes, such as cell survival, growth, migration, and invasion [23,25]. Song and colleagues [122] found a significantly higher miR-155 expression in formalin-fixed paraffin-embedded tumor compared with normal tissues. Multivariate analysis showed an inverse (yet insignificant) correlation with breast cancer outcome in terms of OS. Recently, Kong and colleagues [123] verified these findings and reported miR-155 to be frequently upregulated in various types of cancer where it plays pro-angiogenic, proliferative, and migratory roles. Moreover, they noticed miR-155 expression levels to be associated with poor prognosis in terms of OS [123].

## MicroRNA biogenesis

**Figure 2:**
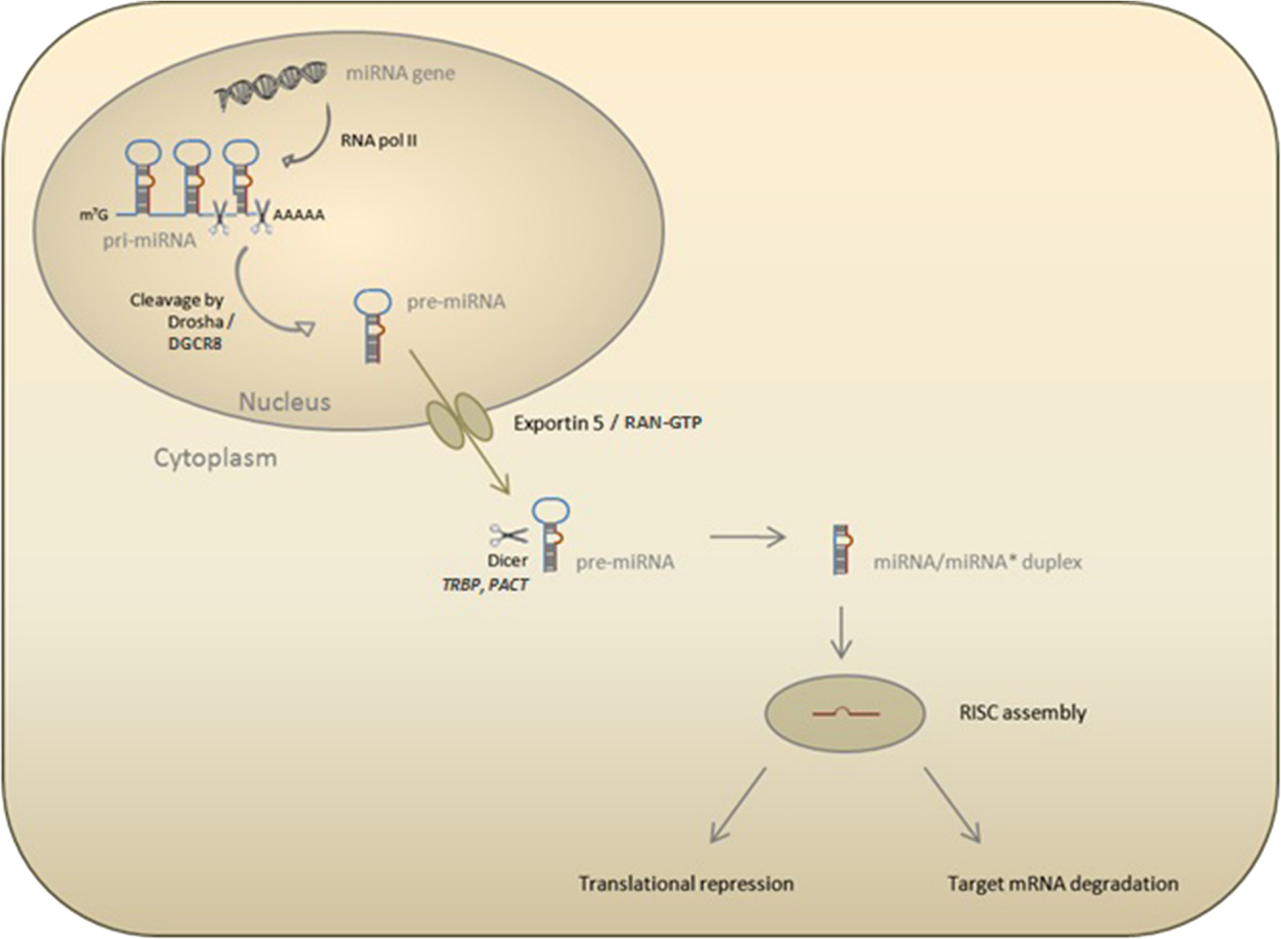
**The microRNA biogenesis pathway.** The canonical biogenesis pathway starts in the nucleus by cleavage of the pri-miRNA transcript by the Drosha-DGCR8 microprocessor complex [128]. After nuclear processing, the resulting precursor hairpin (pre-miRNA) is transported into the cytoplasm by Exportin-5 in complex with Ran-GTP [129]. In the cytoplasm, cleavage into an approximately 22-nucleotide duplex is achieved by the RNase III Dicer [130] and its interactors TRBP and PACT [131,132]. To form the active RNA-induced silencing complex (RISC) that performs gene silencing, the functional guide strand has to be separated from the passenger strand, which is degraded subsequently [133]. miRNA, microRNA.

MiRNA biogenesis (see Figure 2 for the canonical pathway of miRNA biogenesis) affects both the quantity and quality of miRNAs, providing a link between biogenesis and the diagnostic, predictive, and prognostic power of miRNAs.

### MicroRNA biogenesis and the diagnostic potential of microRNAs

A differential expression of miRNA-processing enzymes may result in an altered turnover of pri- and pre-miRNAs, which may influence the biology and etiology of cancer. We [68] and others [69] have seen that the expression of miRNA biogenesis genes varies between different molecular subtypes. Therefore, the hypothesis exists that molecular changes in miRNA-processing enzymes might be responsible for the subtype-specific variation of miRNA profiles.

Blenkiron and colleagues [69] demonstrated how DICER1 and AGO2 showed a significantly lower and respectively higher expression in the more aggressive subtypes. Higher AGO2 and DROSHA levels and lower DICER1 levels were detected in ER-negative breast cancers.

### MicroRNA biogenesis and the predictive and prognostic potential of microRNAs

Aside from affecting the quantity, the miRNA biogenesis cascade may also affect the quality and function of miRNAs. MiRNAs exert their function in collaboration with various proteins in the RISC complex, such as the processing enzymes AGO2, DICER1, and TARBP2. When overexpression of a certain miRNA results in a predictive or prognostic potential, this property could be negated by inadequate expression of AGO2, the catalytic subunit of RISC. Therefore, the prognostic or predictive capability of certain miRNAs may depend on the status of particular miRNA-processing enzymes, which may in fact explain the discrepancy found between several studies.

The role of miRNA-processing enzymes in breast cancer prognosis was also examined in different studies. For example, lower DICER1 levels are significantly associated with lower metastasis and DFS rates [124]. In terms of predictive potential, a loss of Drosha was independently associated with better response to chemotherapeutic and endocrine therapy [125]. In a case-control study, Leaderer and colleagues [126] reported how higher methylation levels in the XPO5 promoter region were associated with reduced risk of breast cancer. Finally, Sung and colleagues [127] reported seven single-nucleotide polymorphisms in miRNA biogenesis genes to be significantly associated with breast cancer survival.

## Conclusions

Over recent years, several research groups have explored the regulatory potential of the miRNA class of small non-coding RNAs. Differences in expression of these miRNAs were used to explain the heterogeneity and disease pathology of several types of cancer. Aside from understanding the biochemical mechanisms these molecules have in gene regulation, we are beginning to understand the carcinogenic mechanisms and pathways these small molecules could influence. Due to their obvious association with carcinogenesis, and considering their promising diagnostic and therapeutic potential, miRNAs will steadily be investigated to discover their effect on a patient’s disease.

Additionally, miRNAs represent a novel class of potential biomarkers for diagnosis, prediction of treatment, or prognosis, which are essential to improve patient management. In this review, we focused on miRNAs validated by more than one study or where results rely on preclinical as well as clinical evidence. However, it needs to be emphasized that the major challenge in identifying diagnostic, predictive, and prognostic miRNAs still resides in the need to firmly validate these findings in additional independent cohorts or by additional preclinical/clinical verification studies. Also, it is important to integrate multivariate testing since many individual identified miRNAs lose their significance when correcting for multiple testing. One should also take into account that some inconsistencies can be partly attributed to differences in subtype distribution or cells of origin. In this way, sufficient experimental evidence can be obtained in order to provide more power to the currently insufficiently strong miRNA markers so that they could be employed in clinical decision-making.

## Additional file

Additional file 1: Table S1. Predictive microRNAs - microRNAs involved in response (sensitivity/resistance) to conventional breast cancer therapeutic strategies. **Table S2A.** Prognostic microRNAs: list of major positive prognostic microRNA signatures in breast cancer. **Table S2B.** Prognostic microRNAs: list of major negative prognostic microRNA signatures in breast cancer.

## Abbreviations

BRCA1: Breast cancer 1, early onset
Cx43: Connexin 43
DFS: Disease-free survival
EMT: Epithelial-to-mesenchymal transition
EPO: Erythropoietin
ER: Estrogen receptor
ERα: Estrogen receptor alpha
FOXO3: Forkhead box protein 3
HIF1A: Hypoxia-inducible factor 1 α
HMGB3: High mobility group box 3 gene
HOXD10: Homeobox D10
IBC: Inflammatory breast cancer
IDC: Invasive ductal carcinoma
IL: Interleukin
LR: Local recurrence
miRNA: microRNA
MTDH: Metadherin
NCT: Neoadjuvant chemotherapy
NF-κB: Nuclear factor-kappa-B
OS: Overall survival
PARP: Poly (ADP-ribose) polymerase
PR: Progesterone receptor
qRT-PCR: Quantitative real-time polymerase chain reaction
RFS: Relapse-free survival
RhoA: Ras homolog gene family
RISC: RNA-induced silencing complex
RT-PCR: Real-time polymerase chain reaction
SOCS1: Suppressor of cytokine signaling 1
SOX4: SRY-related HMG-box4
TNBC: Triple-negative breast cancer
TNC: Tenascin C
UTR: Untranslated region
WAVE3: WAS protein family, member 3
