# Supplementary material for: Dysregulation of microRNAs in breast cancer and their potential role as prognostic and predictive biomarkers in patient management
Source: Breast Cancer Res. 2015 Feb 18;17:21. doi: 10.1186/s13058-015-0526-y (PMC4332424; doi:10.1186/s13058-015-0526-y)
Supplement: Additional file 1: Table S1. — Predictive microRNAs - microRNAs involved in response (sensitivity/resistance) to conventional breast cancer therapeutic strategies. Table S2A. Prognostic microRNAs: list of major positive prognostic microRNA signatures in breast cancer. Table S2B. Prognostic microRNAs: list of major negative prognostic microRNA signatures in breast cancer. [file 13058_2015_526_MOESM1_ESM.doc]

**Additional file 1**

**Table S1. Predictive microRNAs - microRNAs involved in response (sensitivity/resistance) to conventional breast cancer therapeutic strategies**

| Therapy | Generic name | miRNA | miR target | Role in response^a^ | Evidence | Number of patients or type of cells | Remarks | Reference |
| --- | --- | --- | --- | --- | --- | --- | --- | --- |
| Hormone therapy |  |  |  |  |  |  |  |  |
| SERM | Tamoxifen | miR-375 | MTDH | Sensitivity | Preclinical/  clinical | 2 BC datasets | Clinical and preclinical, validated | [80] |
|  |  | miR-342 | BMP7, GEMIN4 | Sensitivity | Preclinical | MCF-7 | Clinical and preclinical, validated in >1 study | [74] |
|  |  |  |  | Sensitivity | Clinical | n = 791 |  | [81] |
|  |  | miR-221/222 | ERα, TIMP3 | Resistance | Preclinical | MCF-7, T47D, MM-468 | Validated in >1 study, clinical evidence required | [82-84] |
|  |  | miR-30c | NR | Sensitivity | Clinical | n = 246 | No preclinical evidence or validation | Rodríguez-González *et al*., *Breast Cancer Res Treat* 2011, 127:43-45 |
|  |  | miR-15a/16 | BCL2 | Sensitivity | Preclinical | MCF-7 | No clinical evidence or validation | Cittelly *et al*., *Carcinogenesis* 2010, 31:2049-2057 |
| SERD | Fulvestrant | miR-221/222 | p27Kip1, ERα | Resistance | Preclinical | MCF-7 | Validated in >1 study, clinical evidence required | [85] |
| Aromatase inhibitor | Letrozole | let-7f | CYP19A1 | Sensitivity | Preclinical/  clinical | n = 23 | Clinical and preclinical evidence | [86] |
| Targeted therapy |  |  |  |  |  |  |  |  |
| Monoclonal antibody | Trastuzumab | miR-210 | MET, IGF1R | Resistance | Preclinical/  clinical | n = 43 | Clinical and preclinical evidence | [87] |
|  |  | miR-200c | ZNF217, ZEB1 | Sensitivity | Preclinical | SKBR3 | No preclinical evidence or validation | Bai *et al*., *Int J Cancer* 2014, 135:1356-1368 |
|  |  | miR-221 | PTEN | Resistance | Preclinical | SKBR3 | No preclinical evidence or validation | Ye *et al*., *BMB Rep* 2014, 47:268-273 |
|  |  | miR-375 | IGF1R | Sensitivity | Preclinical | SKBR3 | No preclinical evidence or validation | Ye *et al*., *BMC Cancer* 2014, 14:134 |
| Tyrosine kinase inhibitor | Gefitinib, Lapatinib | miR-205 | HER3-R | Sensitivity | Preclinical | SKBR3 | No preclinical evidence or validation | Iorio *et al*., *Cancer Res* 2009, 69:2195-2200 |
| Small molecule | Olaparib | miR-182 | BRCA1 | Sensitivity | Preclinical | HL60, K562, MCF7 | No preclinical evidence or validation | Moskwa *et al*., *Mol Cell* 2011, 41:210-220 |
| Chemotherapy |  |  |  |  |  |  |  |  |
|  | FEC | miR-125b | E2F3 | Resistance | Preclinical/  clinical | n = 56 | Clinical and preclinical, validated in >1 study | [89] |
|  |  |  | BCL2 | Resistance | Preclinical | MM-435, SKBR3 | Clinical and preclinical, validated in >1 study | [88] |
|  |  |  | ETS1 | Resistance | Clinical | n = 185 | Clinical and preclinical, validated in >1 study | [90] |
|  | Taxol/Doxo | miR-30c | TWF1, VIM, YWHAZ | Sensitivity | Preclinical | T47D, MCF-7, MM-231 | Validated in >1 study, clinical evidence required | [91,92] |
|  | Taxol | miR-21 | PDCD4, Bcl-2 (up) | Resistance | Preclinical | MM-468 | Validated in >1 study, clinical evidence required | [9,93] |
|  | Epirubicin/Doxo/Taxol | miR-221 | p27Kip1 | Resistance | Clinical | n = 93 | No preclinical evidence or validation | Zhao *et al*., *Onkologie* 2011, 34:675-680 |
| Radiotherapy |  |  |  |  |  |  |  |  |
|  | Radiotherapy | miR-34a | Bcl-2, Notch1, Cycl.D1 | Sensitivity | Preclinical | T47D, MCF-7, MM-231 | Validated in >1 study, clinical evidence required | [95,96] |
|  | Radiotherapy | miR-302 | AKT1, RAD52 | Sensitivity | Preclinical | MM-231, SKBR3 | No clinical evidence or validation | Liang *et al*., *Pharm Res* 2013, 30:1008-1016 |

^a^MicroRNA (miRNA) overexpression leads to an increase in resistance or sensitivity to the mentioned therapy (referred to as ‘resistance’ or ‘sensitivity’, respectively). AKT1, protein kinase B; BC, breast cancer; BCL2, B-cell CLL/lymphoma 2; BMP7, bone morphogenetic protein 7; BRCA1, breast cancer 1, early onset; CYP19A1, aromatase; Doxo, doxorubicin; ERα, estrogen receptor alpha; ETS1, protein C-ets-1; FEC, fluorouracil, epirubucin, and cyclophosphamide; GEMIN4, gem (nuclear organelle)-associated protein 4; IGF1R, insulin-like growth factor 1 receptor; MET, hepatocyte growth factor receptor; MTDH, metadherin; NR, not reported; PDCD4, programmed cell death 4; PTEN, phosphatase and tensin homolog; RAD52, RAD52 homolog; SERD, selective estrogen receptor downregulator; SERM, selective estrogen receptor modulator; Taxol, paclitaxel; TIMP3, tissue inhibitor of metalloproteinase-3; TWF1, twinfilin actin-binding protein 1; VIM, vimentin; YWHAZ, tyrosine 3-monooxygenase/tryptophan 5-monooxygenase activation protein zeta; ZEB1, zinc finger E-box-binding homeobox 1; ZNF217, zinc finger protein 217.

**Table S2A. Prognostic microRNAs: list of major positive prognostic microRNA signatures in breast cancer**

| miRNA | Detection | Type of evidence | Prognostic value | Statistics | | | Validation | | Cells or origin | Reference |
| --- | --- | --- | --- | --- | --- | --- | --- | --- | --- | --- |
|  |  |  |  | Method | Results | Number of samples | Technique | Number of samples |  |  |
| let-7b | LNA-ISH | Clinical | Survival | MV analysis | HR (95% CI): 0.79 (0.65-0.97) | 1,432 TMAs | qRT-PCR | 40 | Early invasive BC | [97] |
|  |  |  |  |  | *P* value: 0.02 |  |  |  |  |  |
|  |  |  |  |  | Covariates: grade, size, LN |  |  |  |  |  |
|  | LNA-ISH | Clinical | OS, RFS | Kaplan Meier analysis | OS time, months (95% CI): 59.8 (54.8-64.8) for high let-7b expression and 54.0 (50.7–57.3) for low let-7b expression | 80 (28 with high and 52 with low let-7b expression) | NR | NR | Heterogeneous BC cohort | [101] |
|  |  |  |  |  | *P* value: 0.027 |  |  |  |  |  |
|  | LNA-ISH | Clinical | OS, RFS | Kaplan Meier analysis | RFS time, months (95% CI): 56.3 (49.4-63.6) for high let-7b expression and 46.1 (40.9–51.4) for low let-7b expression | 80 (28 with high and 52 with low let-7b expression) | NR | NR | Heterogeneous BC cohort | [101] |
|  |  |  |  |  | *P* value: 0.016 |  |  |  |  |  |
| miR-205 | LNA-ISH | Clinical | Survival | MV analysis | HR (95% CI): 0.77 (0.62-0.96) | 1,475 | qRT-PCR | 40 | Ductal BC | [97] |
|  |  |  |  |  | *P* value: 0.02 |  |  |  |  |  |
|  |  |  |  |  | Covariates: grade, size |  |  |  |  |  |
|  | qRT-PCR | Clinical | Disease-free interval | MV analysis | HR (95% CI): 0.50 (0.26-0.94) | 84 | NR | NR | Heterogeneous BC cohort | [102] |
|  |  |  |  |  | *P* value: 0.03 |  |  |  |  |  |
|  |  |  |  |  | Covariates: LN, ER/PR status, grade |  |  |  |  |  |
|  |  |  | OS | MV analysis | HR (95% CI): 0.41 (0.18-0.94) | 84 | NR | NR | Heterogeneous BC cohort | [102] |
|  |  |  |  |  | *P* value: 0.036 |  |  |  |  |  |
|  |  |  |  |  | Covariates: LN, ER/PR status |  |  |  |  |  |
| miR-375 | Solexa deep sequencing | Clinical | Metastatic relapse | FC expression miRNA (relapse vs non relapse) | FC: -1.9 | 42 | qRT-PCR | 26 | Stage II-III BC | [57] |
|  |  |  |  |  | *P* value: 5.89E-05 |  |  |  |  |  |
| miR-30a | miRNA microarray | Preclinical/ clinical | Recurrence-free survival | MV analysis | HR (95% CI): 0.25 (0.08-0.82) | 221 | NR | NR | IDC | [104] |
|  |  |  |  |  | *P* value: 0.02 |  |  |  | Heterogeneous BC cohort | [108] |
|  |  |  |  |  | Covariates: stage, ER status |  |  |  |  |  |
|  |  |  | Disease-free survival | MV analysis | HR (95% CI): 0.52 (0.25-1.09) | 221 | NR | NR | IDC | [104] |
|  |  |  |  |  | *P* value: 0.08 |  |  |  | Heterogeneous BC cohort | [108] |
|  |  |  |  |  | Covariates: stage, ER status |  |  |  |  |  |
| miR-34a | miRNA microarray | Clinical | Metastasis-free survival | MV analysis | HR (95% CI): 0.63 (0.41-0.96) | 183 | NR | NR | Heterogeneous BC cohort | Peurala *et al.*, *PLoS One* 2011, 6:e26122. |
|  |  |  |  |  | *P* value: 0.031 |  |  |  |  |  |
|  |  |  |  |  | Covariates: TNM, grade, ER, PR, HER2, p53, Ki67 |  |  |  |  |  |
| miR-133a | ISH | Clinical | RFS | Kaplan Meier analysis | RFS time, months: 56.6 (patients with miR-133a expression) and 45.5 (patients without miR-133a expression) | 90 (66 with and 24 without miR-133a expression) | NR | NR | Heterogeneous BC cohort | Wu *et al., BMC Cancer* 2012, 12:51. |
|  |  |  |  |  | *P* value: 0.04 |  |  |  |  |  |
| miR-195 | qRT-PCR | Clinical | Recurrence-free survival and OS | MV analysis | HR (95% CI): 0.04 (0.009-0.179) | 88 | NR | NR | Heterogeneous BC cohort | Song CG *et al., Zhonghua Wai Ke Za Zhi* 2012, 50:353-356. |
|  |  |  |  |  | *P* value: 0.000 |  |  |  |  |  |
|  |  |  |  |  | Covariates: NR |  |  |  |  |  |
| miR-342-5p | miRNA microarray | Clinical | Recurrence-free survival | Kaplan Meier analysis | High miR-342-5p: higher time to LR | 101 | miRNA microarray | 1302 | Heterogeneous BC cohort | [109] |
|  |  |  |  |  | *P* value: 0.037 |  |  |  |  |  |
| miR-497 | qRT-PCR | Clinical | Disease-free survival | MV analysis | HR (95% CI): 0.65 (0.43-0.89) | 128 | NR | NR | Heterogeneous BC cohort | [111] |
|  |  |  |  |  | *P* value: 0.016 |  |  |  |  |  |
|  |  |  |  |  | Covariates: size, grade, stage |  |  |  |  |  |
|  |  |  | OS | MV analysis | HR (95% CI): 0.47 (0.33-0.54) | 128 | NR | NR | Heterogeneous BC cohort | [111] |
|  |  |  |  |  | *P* value: 0.008 |  |  |  | IDC | [110] |
|  |  |  |  |  | Covariates: size, grade, stage |  |  |  |  |  |
| miR-324-5p | RT-PCR | Clinical | Metastasis-free survival | MV analysis | HR (95% CI): 0.31 (0.07-0.79) | 12 IBC, 32 non-IBC | NR | NR | IBC and non-IBC | Lerebours *et al., Int J Cancer* 2013, 133:1614-1623. |
|  |  |  |  |  | *P* value: 0.01 |  |  |  |  |  |
|  |  |  |  |  | Covariates: size, LN, grade, stage, ER, PR, HER2 status |  |  |  |  |  |

BC, breast cancer; CI, confidence interval; ER, estrogen receptor; FC, fold change; HR, hazard ratio; IBC, inflammatory breast cancer; IDC, invasive ductal carcinoma; ISH, *in situ* hybridization; LN, lymph node; LNA, Locked Nucleic Acid; LR, local recurrence; miRNA, microRNA; MV, multivariate; NR, not reported; OS, overall survival; PR, progesterone receptor; qRT-PCR, quantitative real-time polymerase chain reaction; RFS, relapse-free survival; TMA, transcription-mediated amplification; TNM, tumor, node, metastasis.

**Table S2B. Prognostic microRNAs: list of major negative prognostic microRNA signatures in breast cancer**

| miRNA | Detection | Type of evidence | Prognostic value | Statistics | | | Validation | | Cells or origin | References |
| --- | --- | --- | --- | --- | --- | --- | --- | --- | --- | --- |
|  |  |  |  | Method | Results | Number of samples | Technique | Number of samples |  |  |
| miR-34b | qRT-PCR | Clinical | Disease-free survival | Kaplan-Meier and log rank | Positive correlation miR-34b expression and DFS | 39 | NR | NR | TNBC | Svoboda *et al., Diagn Pathol* 2012, 7:31. |
|  |  |  |  |  | *P* value: 0.0020 |  |  |  |  |  |
|  |  |  | OS | Kaplan-Meier and log rank | Positive correlation miR-34b expression and OS | 39 | NR | NR | TNBC | Svoboda *et al., Diagn Pathol* 2012, 7:31. |
|  |  |  |  |  | *P* value: 0.0008 |  |  |  |  |  |
| miR-122 | Solexa deep sequencing | Clinical | Metastatic relapse | FC expression miRNA (relapse vs non relapse) | FC: 1.35 | 42 | qRT-PCR | 26 | Heterogeneous BC cohort | [57] |
|  |  |  |  |  | *P* value: 2.98E-05 |  |  |  |  |  |
| miR-27b-3p | qRT-PCR | Clinical | Distant metastasis-free survival | MV analysis | HR (95% CI): 6.651 (1.24-35.69) | 58 | qRT-PCR | 41 | TNBC | [113] |
|  |  |  |  |  | *P* value: 0.027 |  |  |  |  |  |
|  |  |  |  |  | Covariates: LN, miR-107, miR-103a-3p |  |  |  |  |  |
| miR-21 | qRT-PCR | Clinical | OS | MV analysis | HR (95% CI): 14.21 (1.34-15.10) | 109 | NR | NR | IDC | [115] |
|  |  |  |  |  | *P* value: 0.028 |  |  |  |  |  |
|  |  |  |  |  | Covariates: size, ER status |  |  |  |  |  |
|  | qRT-PCR | Clinical | Disease-free survival | MV analysis | HR (95% CI): 2.49 (1.30-4.80) | 84 | NR | NR | Heterogeneous BC cohort | [102] |
|  |  |  |  |  | *P* value: 0.006 |  |  |  |  |  |
|  |  |  |  |  | Covariates: LN, ER/PR status, grade |  |  |  |  |  |
| miR-210 | Deep sequencing | Clinical | Time to metastasis | MV analysis | HR (95% CI): 1.39 (NR) | 80 | NR | NR | IDC | [75] |
|  |  |  |  |  | *P* value: 0.018 |  |  |  |  |  |
|  |  |  |  |  | Covariates: NR |  |  |  |  |  |
|  |  |  | OS | MV analysis | HR (95% CI): 1.54 (NR) | 80 | NR | NR | IDC | [75] |
|  |  |  |  |  | *P* value: 0.004 |  |  |  |  |  |
|  |  |  |  |  | Covariates: NR |  |  |  |  |  |
|  | Meta-analysis |  | Recurrence-free survival | MV analysis | HR (95% CI): 2.47 (1.36-4.46) | 699 | Meta-analysis | Meta-analysis | Meta-analysis | [116] |
|  |  |  |  |  | *P* value: 0.004 |  |  |  |  |  |
|  |  |  |  |  | Covariates: NR |  |  |  |  |  |
|  | Meta-analysis |  | Disease-free survival | MV analysis | HR (95% CI): 1.89 (1.30-2.74) | 1,809 | Meta-analysis | Meta-analysis | Meta-analysis | [117] |
|  |  |  |  |  | *P* value: 0.001 |  |  |  |  |  |
|  |  |  |  |  | Covariates: NR |  |  |  |  |  |
|  |  |  | Progression-free survival | MV analysis | HR (95% CI): 1.20 (1.05-1.38) | 1,809 | Meta-analysis | Meta-analysis | Meta-analysis | [117] |
|  |  |  |  |  | *P* value: 0.007 |  |  |  |  |  |
|  |  |  |  |  | Covariates: NR |  |  |  |  |  |
|  |  |  | RFS | MV analysis | HR (95% CI): 4.42 (2.14-9.15) | 1,809 | Meta-analysis | Meta-analysis | Meta-analysis | [117] |
|  |  |  |  |  | *P* value: 0.000 |  |  |  |  |  |
|  |  |  |  |  | Covariates: NR |  |  |  |  |  |
| miR-9 | miRNA microarray | Clinical | LR-free survival | Kaplan Meier analysis | LR-free survival rate: 30.8% (high miR-9 expression) and 67.9% (low miR-9 expression) | 16 | qRT-PCR | 52 | ER-positive BC | [120] |
|  |  |  |  |  | *P* value: 0.02 |  |  |  |  |  |
| miR-187 | LNA miRCURY probe for miR-187 | Preclinical/ clinical | BC-specific survival | MV analysis | HR (95% CI): 7.37 (2.05-26.51) | 117 | LNA miR probe | 470 | Heterogeneous BC cohort | [121] |
|  |  |  |  |  | *P* value: 0.002 |  |  |  |  |  |
|  |  |  |  |  | Covariates: age, size, grade, LN, ER status, PR status, HER2 status |  |  |  |  |  |
|  |  |  | BC-specific survival | MV analysis | HR (95% CI): 13.74 (2.62-72.03) | 46 | LNA miR probe | 156 | LN-positive BC | [121] |
|  |  |  |  |  | *P* value: 0.002 |  |  |  |  |  |
|  |  |  |  |  | Covariates: age, size, grade, LN, ER status, PR status, HER2 status |  |  |  |  |  |
|  |  |  | Recurrence-free survival | MV analysis | HR (95% CI): 1.99 (1.21-3.28) | 462 | NR | NR | Heterogeneous BC cohort | [121] |
|  |  |  |  |  | *P* value: 0.007 |  |  |  |  |  |
|  |  |  |  |  | Covariates: age, size, grade, LN, ER status, PR status, HER2 status |  |  |  |  |  |
|  |  |  | Recurrence-free survival | MV analysis | HR (95% CI): 2.21 (1.18-4.14) | 155 | NR | NR | LN-positive BC | [121] |
|  |  |  |  |  | *P* value: 0.014 |  |  |  |  |  |
|  |  |  |  |  | Covariates: age, size, grade, LN, ER status, PR status, HER2 status |  |  |  |  |  |
| miR-155 | qRT-PCR | Clinical | OS | MV analysis | HR (95% CI): 1.58 (0.87-3.16) | 88 | NR | NR | Heterogeneous BC cohort | [122] |
|  |  |  |  |  | *P* value: 0.082 |  |  |  |  |  |
|  |  |  |  |  | Covariates: NR |  |  |  |  |  |
|  | qRT-PCR | Clinical | OS | MV analysis | HR (95% CI): 2.377 (1.461-3.866) | 231 | NR | NR | Heterogeneous BC cohort | [123] |
|  |  |  |  |  | *P* value: <0.001 |  |  |  |  |  |
|  |  |  |  |  | Covariates: age, stage, grade, histology |  |  |  |  |  |
| miR-221/222 | qRT-PCR | Clinical | Distant metastasis-free survival | MV analysis | Relative risk: 2.57 | 86 | NR | NR | Advanced BC | Falkenberg *et al., Br J Cancer* 2013, 109:2714-2723 |
|  |  |  |  |  | *P* value: 0.028 |  |  |  |  |  |
|  |  |  |  |  | Covariates: size, LN, HER2 |  |  |  |  |  |

BC, breast cancer; CI, confidence interval; ER, estrogen receptor; FC, fold change; HR, hazard ratio; IDC, invasive ductal carcinoma; LN, lymph node; LNA, locked nucleic acid; LR, local recurrence; miRNA, microRNA; MV, multivariate; NR, not reported; OS, overall survival; PR, progesterone receptor; qRT-PCR, quantitative real-time polymerase chain reaction; RFS, relapse-free survival; TNBC, triple-negative breast cancer.
